# Prospective evaluation of real-world performance and clinical impact of the Biofire FilmArray joint infection panel

**DOI:** 10.1128/spectrum.02239-24

**Published:** 2025-02-25

**Authors:** Benjamin Berinson, Konstantin Tanida, Laura Spenke, Lukas Krivec, Johannes Keller, Tim Rolvien, Martin Christner, Marc Lütgehetmann, Martin Aepfelbacher, Till Orla Klatte, Holger Rohde

**Affiliations:** 1 Institute for Medical Microbiology, Virology and Hygiene, University Medical Center Hamburg-Eppendorf, Hamburg, Germany; 2 Department of Trauma and Orthopedic Surgery, University Medical Center Hamburg-Eppendorf, Hamburg, Germany; MultiCare Health System, Tacoma, Washington, USA

**Keywords:** joint infection, synovial fluid, diagnostics, multiplex PCR, FilmArray, species identification, resistance, diagnostic stewardship

## Abstract

Limitations of culture-based diagnostic approaches in pathogen detection in joint infections (JI) can be overcome by amplification-based, molecular assays. Recently, a syndromic panel PCR (spPCR) assay (Biofire JI panel; BJA) was approved for pathogen identification from synovial fluid (SF). Here, the performance and the clinical impact of the BJA were assessed in comparison to standard of care diagnostics in a prospective cohort of patients presenting with symptoms consistent with JI. One hundred sixty-five synovial fluids underwent analysis using the BJA. The results were compared with culture-based diagnostics. Discrepant results were re-analyzed using species-specific PCRs or 16S-rDNA sequencing. Clinical data from patients were collected to evaluate the impact on patient management. Twenty-seven of 165 (16.3%) synovial fluid cultures grew bacterial pathogens. In 24/27 cases, the BJA results were concordant. In one case, the cultured pathogen was missed, but three additional pathogens were identified. In 11 culture-negative cases, BJA identified a pathogen. Mean turnaround time in culture-positive samples was 14:11 h and 35:17 h in BJA and culture, respectively. In 11 cases, antibiotic therapy was optimized, based on BJA results. This study demonstrates high sensitivity and specificity (96.3% and 97.8%, respectively) of BJA, as well as a shorter turnaround time than culture-based techniques (21 h faster). Based on analysis of clinical data, antibiotic therapy was optimized due to BJA results in 11 cases. Care must be taken, as important pathogens in prosthetic JI are not included in the panel, restricting its value here.

**IMPORTANCE:**

Pathogen detection is critical for targeted management of joint infections; however, cultural detection of pathogens can be challenging. The Biofire Joint Infection Assay (BJA) is a syndromic panel PCR test that allows culture-independent detection of 31 pathogens. The diagnostic performance and clinical impact were evaluated in a cohort of 160 patients with native and prosthetic joint infections. BJA detected concordant pathogens in 24 of 27 culture-positive cases and enabled the detection of additional pathogens in 11 patients. The time to result was significantly shorter than with standard culture-based diagnostics (14 vs 35 h), and BJA allowed optimization of therapy in 11 patients. The data show that BJA is a relevant addition to the diagnostic options for joint infections. Limitations result from incomplete detection of relevant pathogens, especially in prosthetic joint infections. The use of BJA in daily practice must therefore be accompanied by diagnostic stewardship measures.

## INTRODUCTION

Joint infections (JI) can be a life-threatening and disabling condition. In Europe, native joint infections (NJI) have a prevalence of four to 10 cases per 100,000 patient-years, with young children and older adults being at the highest risk (1–4). In addition to NJI, prosthetic joint infections (PJI) are also steadily becoming more prevalent. As recently published, there are between 70 and 180 PJI cases per 100,000 patient-years (5), likely due to the increasing number of implanted prosthetic joints in an aging population (6).

It is important to note that there are significant differences in the distribution of bacterial species causing NJI or PJI. NJI typically involves highly virulent pathogens, such as *Staphylococcus aureus*, beta-hemolytic streptococci, and Gram-negative rods, resulting in prominent symptoms and signs of inflammation (2, 5, 7). In contrast, PJI can be caused by various pathogens, including low-virulence organisms such as coagulase-negative staphylococci (CoNS), *Cutibacterium spp*., viridans streptococci, and *Enterococcus spp* (8).

To optimize patient management, identifying the causative pathogen is crucial. This typically relies on classic microbiological culture techniques from synovial fluid (SF) or tissue specimens (9). A significant proportion of NJI and PJI, however, remains culture-negative, for example, related to the presence of fastidious organisms Moreover, sensitivity may be further reduced due to inappropriate storage (i.e., prolonged time until sending the sample to the lab, extended transportation times, inappropriate temperature) of the primary specimen and prior antimicrobial exposure (8, 10–14). Inability to recover the causative pathogen has immediate consequences for patient management, that is, the requirement for using broad-spectrum antibiotics and, in PJI, two-stage revision surgical strategies (12).

These limitations call for more accurate, if possible, culture-independent, approaches. Here, molecular techniques have been brought into play. 16S rDNA PCR assays have shown to be promising but are inherently insensitive and often associated with false-negative results (15). Species-specific PCR assays overcome these limitations with regard to sensitivity, and commercially available syndromic panel assays (i.e., Unyvero, Biofire JI Panel Assay [BJA]) offer a convenient option for broader implementation of molecular approaches, at a higher cost than culture methods and a pre-defined panel of covered organisms. The BJA is able to detect 31 pathogens and eight clinically relevant genetic resistance markers directly from SF. However, the clinical consequences of BJA for clinical management are still under evaluation (11, 13, 16).

The aim of this study was to investigate the clinical usefulness and added value of the BJA to diagnose NJI or PJI in patients admitted to a tertiary-care University Medical Center in Hamburg, Germany. BJA real-life performance was compared with the culture-based standard of care (SOC) in 165 SF specimens from 160 patients. This is a prospective study assessing real-life laboratory performance like turnaround time and evaluating the impact of BJA on patient management and respective antibiotic treatment decisions.

## MATERIALS AND METHODS

### Study setting and inclusion criteria

This prospective single-center study was conducted at the University Medical Center Hamburg-Eppendorf, Germany, a 17,00-bed tertiary care university hospital. The study was performed between September 2022 and September 2023. All SF, sent for routine microbiological analysis (described below), were subjected in the same workflow to the BJA. Exclusion criteria for BJA analysis were follow-up arthrocentesis of the same joint within 30 days, too little sample volume for BJA, and patient age <18 years. In total, 165 specimens from 160 individual patients were included. Of note, five patients had synovial specimens obtained from two different joints or a minimum of 30 days between arthrocentesis. In these patients, each independent joint disease is regarded as a case by itself. Data from these 165 cases were analyzed for impact of BJA results on clinical patient management.

### Conventional synovial fluid analysis (SOC)

In total, 50 µL of synovial fluid was subjected to a Gram stain, employing a PolyStainer (IUL Instruments). For the culture of aerobic bacteria and yeast, 50 µL of SF was spread onto Columbia sheep blood agar, chocolate agar, and Sabouraud agar (all from Oxoid, Basingstoke, UK); 50 µL was streaked onto Schaedler anaerobic agar (Oxoid, Basingstoke, UK) for the cultivation of anaerobic bacteria. Incubation was carried out at 37°C and 5% CO_2_ for up to 14 days or anaerobic conditions for 48 h. Plates were evaluated for growth after 24 h, 48 h, 7 days, and 14 days. In addition to solid media, 2 mL of an enrichment thioglycolate broth was inoculated with up to 500 µL of the SF and incubated at 37°C for 2 days. Cultivated microorganisms were identified to the species level using a Maldi-ToF instrument (Microflex, Bruker Daltonics, Bremen, Germany).

A VITEK 2 instrument (bioMérieux, Marcy l´Étoile, France) was used for susceptibility testing, using either the VITEK 2 AST-N223 (*Enterobacterales*) or the VITEK AST-P611 card (staphylococci, enterococci). Agar diffusion was employed to test fastidious organisms (e.g., streptococci) according to EUCAST protocols.

### Biofire JI analysis

BJA was performed according to the manufacturer’s guidelines. In short, a provided hydration solution was applied to the test pouch, and 200 µL of synovial fluid was mixed with the sample buffer of the kit. This was added to the pouch, which was then loaded into the instrument. Nucleic acid extraction, a multiplexed nested PCR, and a product melt temperature analysis were performed by the Biofire instrument. An overview of genera or families of bacteria and resistance markers represented on BJA in the here-utilized version is shown in the supplements (Table S1).

### Verification of discrepant results

When discrepant results between culture and BJA were encountered and if synovial fluid specimens were still available, a rerun of the BJA was performed (*n* = 7). Furthermore, if additional SF specimens were available and in-house PCRs were already established, respective samples were subjected to species-specific PCRs or to a CE-IVD-certified 16S rDNA sequencing analysis workflow (Molzym; Bremen, Germany) if no in-house PCRs were available. For species-specific PCRs, nucleic acids were extracted from frozen original specimens using a MagNa Pure 96 System (Roche, Mannheim, Germany), and PCR was performed on a LightCycler 480 II instrument (Roche, Mannheim, Germany). Primers and probes were used as published previously (*S. aureus* and *S. agalactiae*, both described by Diaz et al. (17)]. 16S rDNA sequencing was performed as previously described (18). In brief, 1 mL of the homogenized specimen was depleted of human DNA, and bacterial DNA was isolated. After a PCR on a LightCycler 480 II utilizing the Mastermix 16S complete (Molzym; Bremen, Germany), a manual analysis of amplification and melt curves was performed. Samples with PCR amplification curves with a Ct-value <35 and a T_m_ between 86°C and 92°C were regarded as positive. Amplicons were purified and sent for Sanger sequencing to an external provider (Microsynth Seqlab GmbH, Goettingen, Germany). 16S rDNA sequences were analyzed using the web-based SepsiTest-BLAST application provided by Molzym. Additionally, the results were verified using basic local alignment search tool (BLAST) by National Center for Biotechnology Information (NCBI).

### Clinical impact evaluation

To study the impact on clinical management, patients were assigned to four clinical categories: NJI, PJI, inflammatory non-infectious joint disease (i.e., gout), or non-infectious non-inflammatory joint disease. PJI and NJI were diagnosed clinically (i.e., clinical presentation, increased C-reactive protein [CRP], and increased leukocyte count in joint aspirate). The responsible microbiologist called the corresponding ward with either a positive BJA result or after a species has been identified via SOC. Data were analyzed with regard to patients receiving optimal, adequate, or inadequate treatment. Optimal treatment was defined as the use of an antimicrobial substance with the narrowest possible spectrum of action, taking into account indications and contraindications (according to local and international recommendations for the treatment of infectious diseases). Adequate antimicrobial therapy was defined as the use of an antimicrobial for which *in vitro* activity against the infecting microorganism was proven, whereas inadequate therapy was defined if the identified organism was not covered. De-escalation was defined as a change from a broad-spectrum antimicrobial therapy to an antibiotic with less broad-spectrum coverage, whereas escalation was defined as a change to a broad-spectrum antimicrobial therapy from an antibiotic with less broad-spectrum coverage. Total turnaround time (TTAT) was evaluated for culture-positive samples. TTAT was defined as the time between the sample arriving at the lab and the first generated report, with BJA results and/or species identification via culture, which was made available to the clinicians.

The following data were recorded by chart review if available: demographics, native or prosthetic joint, synovial fluid analysis (cell count, neutrophil percentage), C-reactive protein in serum, histopathologic findings, and duration of the antimicrobial treatment administered before and after result of BJA and SOC (empirical and targeted treatment).

### Quality control

The quality control (QC) for the matrix-assisted laser desorption/ionization time of flight (MALDI-ToF) analysis was performed on a daily basis with *Escherichia coli* ATCC 25922 and *Candida albicans* ATCC 25922. The EUCAST and VITEK 2 quality control procedures were performed regularly once per week, as previously described (19). Full process control for the species-specific PCRs was performed by an internal spike-in control, which was added during DNA extraction (Cobas omni optimization reagent, Roche). Additionally, the JI assay QC was performed once with the supplied positive and negative QC test vials.

### Statistics

Sensitivity and specificity with corresponding 95% CI were calculated using the MedCalc “Diagnostic Test evaluation calculator” (https://www.medcalc.org/calc/diagnostic_test.php). The results were interpreted as true positives, if SOC showed culture growth of the same species, other methods (see above) confirmed findings of BJA or—if the material was not archived/insufficient volume was present for follow-up testing—the same species was recovered from the same joint (via, i.e., surgical intervention) or in the bloodstream, or previous findings of the same pathogen are recorded in the patient’s chart (i.e., previous SF analysis in a different hospital). Otherwise, the BJA results are interpreted as inconclusive and therefore not counted as a true positive.

## RESULTS

### Study population

165 SF specimens from 160 patients were included in the analysis (one specimen/patient, *n* = 155; two specimens per patient [minimum 30 days between SF aspiration], *n* = 5 patients). The mean patient age was 63.1 years (range: 19–91 years; SD: 16.6). The majority of the SF specimens were obtained from knees (*n* = 118), followed by hips, shoulder, elbow, ankle, and unspecified source of origin (*n* = 30; *n* = 7; *n* = 5; *n* = 2, *n* = 3, respectively). Ninety-four of 165 (57.0%) SF specimens were aspirated from native joints and 71 (43%) from prosthetic joints. Final diagnosis according to the clinical chart review was NJI (*n* = 29; 17.6%), PJI (*n* = 24; 14.5%), inflammatory, non-infectious joint disease (*n* = 30; 18.2%) and non-infectious, non-inflammatory joint disease (*n* = 81; 49.1%), and unspecified (*n* = 1; 0.6%). Further information is available in Table S2.

### Per-specimen analysis

Twenty-seven of 165 (16.3%) specimens exhibited growth on solid agar media (Table 1; 12/94 and 15/71 SF from native joint and prosthetic joints, respectively). No additional pathogen was identified by broth enrichment; 138/165 (83,6%) specimens remained culture-negative. An overview of all culture results is provided in Table S3. Overall, BJA was positive in 38/165 (23.0%) specimens, including all 27 SOC-positive samples. In 24 specimens, species identified by BJA were identical to those recovered by SOC, whereas in three specimens, BJA and SOC gave discrepant results. In one case (case 113), BJA identified an additional pathogen (*Peptinophilus species*). A rerun revealed the same pathogen combination, but this could not be further investigated due to too little sample volume. In case 37, three pathogens were identified by SOC and the BJA (*E. coli*, *Enterococcus faecalis*, and *Citrobacter amalonaticus*), but BJA missed *C. amalonaticus*, being an off-panel organism. Additionally, in case 13, SOC only identified *Pseudomonas aeruginosa*, whereas BJA identified *Finegoldia magna*, *Staphylococcus aureus,* and *Anaerococcus prevotii/vaginali*s. A rerun showed the same result. Interestingly, in this case, several intraoperative tissue samples were gained before and after synovial fluid aspiration, which exhibited several times the (additional) growth of *S. aureus* and once *F. magna*. The sensitivity of the BJA for on-panel pathogens was 96.3% (95% CI: 81.0%–99.9%), and the specificity was 97.8% (95% CI: 93.8%–99.6%). The BJA pathogen coverage rate in this study was 92.6%. No resistance markers were detected by BJA, which is consistent with AST results obtained with SOC approaches. Median TTAT for species identification in samples that were positive by culture was 35:17 h (SD: 12:12 h; range 16:51 h-69:47 h) for SOC, whereas the TTAT for BJA was 14:11 h (SD 2:03 h; range 2:30 h-51:28 h).

**TABLE 1:** Overview of species identification and information about antibiotic therapy in SOC- and BJA-positive samples^*a*^

Case number	Joint type	Diagnosis according to final medical report	Gram stain result	Species identified in SF			Antibiotic therapy^*c*^			
				SOC		BJA	Before sample acquisition	After sample acquisition	Empiric therapy	Adapted therapy^*d*^
				Semi-quantification of growth^*b*^	Species ID					
2	Prosthetic	PJI	Negative	+	*E. faecalis*	*E. faecalis*	0	0	n.a.	n.a.
13	Native	NJI	Negative	(+)	*P. aeruginosa*	*F. magna, S. aureus, A. prevotii/vaginalis*	1	1	Cefazolin	n.a.
23	Prosthetic	PJI	Gram-negative rods	+++	*E. coli*	*E. coli*	1	1	Ceftriaxone and metronidazole	n.a.
30	Prosthetic	PJI	Negative	+++	*E. faecalis*	*E. faecalis*	0	1	Ampicillin/sulbactam	Ampicillin
31	Prosthetic	PJI	Negative	+	*S. aureus*	*S. aureus*	0	1	Vancomycin and ceftriaxone	Flucloxacillin
33	Native	NJI	Negative	+	*S. agalactiae*	*S. agalactiae*	0	1	Ampicillin/sulbactam	Penicillin G^*e*^
35	Native	NJI	Negative	(+)	*S. sanguinis* Group	*Streptococcus spp*.	0	1	Penicillin G	Ceftriaxone
37	Prosthetic	PJI	Gram-positive cocci and Gram-negative rods	++; +++; +++	*E. coli, E. faecalis, C. amalonaticus*	*E. coli, E. faecalis*	0	1	Piperacillin/tazobactam	Ceftriaxone and Ampicillin
44	Native	NJI	Negative	(+)	*S. pneumoniae*	*S. pneumoniae*	1	1	Cefazolin	Ceftriaxone^*e*^
56	Native	NJI	Gram-positive cocci	+++	*S. agalactiae*	*S. agalactiae*	0	1	Vancomycin	Penicillin G
58	Native	NJI	Negative	+	*S. agalactiae*	*S. agalactiae*	1	1	Penicillin G	n.a.
68	Native	NJI	Negative	+++	*S. aureus*	*S. aureus*	0	1	Cefazolin	n.a.
78	Prosthetic	PJI	Gram-positive cocci	+++	*S. aureus*	*S. aureus*	0	1	Flucloxacillin^*e*^	n.a.
79	Prosthetic	PJI	Gram-positive cocci	+++	*S. aureus*	*S. aureus*	0	1	Flucloxacillin	n.a.
80	Prosthetic	PJI	negative	+++	*S. agalactiae*	*S. agalactiae*	0	1	Ceftriaxone and vancomycin	Clindamycin
109	Prosthetic	PJI	Negative	+	*C. koseri*	*Citrobacter spp*.	1	1	Ceftriaxone	n.a.
110	Native	NJI	Gram-negative rods	++	*K. pneumoniae* Group	*K. pneumoniae* Group	0	1	Vancomycin, meropenem, and clindamycin	Ceftriaxone
113	Native	NJI	Gram-positive cocci	++	*S. dysgalactiae/canis*	*Streptococcus spp., Peptinophilus spp*.	1	1	Ampicillin/sulbactam and clindamycin	Meropenem and Clindamycin
125	Prosthetic	PJI	Gram-positive cocci	++	*S. agalactiae*	*S. agalactiae*	0	1	Ampicillin/sulbactam	Penicillin G^*e*^
136	Prosthetic	PJI	Negative	++	*E. coli*	*E. coli*	0	1	Vancomycin and ceftriaxone	Ceftriaxone
138	Prosthetic	PJI	Gram-positive cocci	+++	*S. dysgalactiae/canis*	*Streptococcus spp*.	1	1	Vancomycin and meropenem	n.a.
142	Prosthetic	PJI	Negative	+	*E. faecalis*	*E. faecalis*	1	1	Vancomycin and ceftriaxone	Ampicillin
144	Prosthetic	PJI	Gram-positive cocci	+++	*S. agalactiae*	*S. agalactiae*	1	1	Vancomycin and meropenem	Ceftriaxone
147	Native	NJI	Negative	++	*S. aureus*	*S. aureus*	0	1	Flucloxacillin and rifampicin	n.a.
149	Prosthetic	PJI	Negative	(+)	*S. aureus*	*S. aureus*	0	1	Vancomycin, ceftriaxone, and rifampicin	Flucloxacillin and Rifampicin
163	Native	NJI	Negative	++	*S. aureus*	*S. aureus*	0	1	Ampicillin/sulbactam	Flucloxacillin^*e*^
165	Native	NJI	Negative	++	*S. aureus*	*S. aureus*	0	1	Flucloxacillin^*e*^	n.a.

^*a*^ NJI = Native joint infection, PJI= Prosthetic joint infection, n.a. = not applicable.

^*b*^ Growth of up to 10 colonies is depicted as (+), greater than 10 but limited to the first streak is depicted as +, growth until the second streak is depicted as ++, growth until the third streak is depicted as +++.

^*c*^ 0 = no; 1 = yes.

^*d*^ Antibiotic therapy after the availability of BJA/SOC results.

^*e*^ Antibiotic therapy was adapted/initiated before SOC data was available, therefore based on BJA results.

BJA identified pathogens in 11 SOC-negative cases (Table 2). In three cases, species-specific in-house PCR gave identical results, and these were therefore interpreted as true positives (cases 22, 145, and 155). In two cases, no species-specific in-house PCR was available, and 16S rDNA PCR followed by Sanger sequencing was performed (cases 97 and 118). The results obtained were consistent with the BJA results. In six cases, there was insufficient left-over SF to perform confirmatory PCRs. However, in three cases, (cases 55, 140, and 154), a review of medical records revealed previous findings of the pathogen, as defined above. Therefore, these BJA results are interpreted as true positives. Cases 51, 72, and 139 remain inconclusive and were regarded as false positives.

**TABLE 2:** Overview of species identification and information about antibiotic therapy in SOC-negative but BJA-positive samples^*a*^

Case number	Joint type	Diagnosis according to final medical report	Gram stain result	Species identified in SF		Antibiotic therapy^*b*^			
				SOC	BJA	Before sample acquisition	After sample acquisition	Empiric therapy	Adapted therapy^*c*^
22	Prosthetic	PJI	Negative	Sterile	*S. agalactiae*	0	1	Ampicillin/ sulbactam	Penicillin G^*d*^
51	Native	NJI	Negative	Sterile	*S. pneumoniae*	1	1	Piperacillin/ tazobactam	Ampicillin^*d*^
55	Prosthetic	PJI	Negative	Sterile	*S. aureus*	1	1	Flucloxacillin and fosfomycin	n.a.
72	Prosthetic	Non-inflammatory non-infectious joint disease	Negative	Sterile	*Streptococcus spp*.	0	0	n.a.	n.a.
97	Native	Unknown	Negative	Sterile	*E. cloacae* Complex	1	1	Meropenem	n.a.
118	Native	NJI	Negative	Sterile	*Streptococcus spp*.	0	1	Penicillin G^*d*^	n.a.
139	Prosthetic	PJI	Negative	Sterile	*S. pneumoniae*	1	1	Ampicillin/ sulbactam	n.a.
140	Prosthetic	PJI	Negative	Sterile	*C. albicans*	1	1	Fluconazole	n.a.
145	Native	NJI	Negative	Sterile	*S. aureus*	1	1	Ampicillin/ sulbactam	Cefazolin^*d*^
154	Native	NJI	Negative	Sterile	*Streptococcus spp*.	1	1	Penicillin G	Piperacillin/ tazobactam
155	Prosthetic	PJI	Negative	Sterile	*S. aureus*	1	1	Meropenem	Cefazolin^*d*^

^*a*^ NJI = Native joint infection, PJI= Prosthetic joint infection, n.a. = not applicable.

^*b*^ 0 = no, 1 = yes.

^*c*^ Antibiotic therapy after availability of BJA/SOC results.

^*d*^ Antibiotic therapy was adapted, whereas SOC remained negative, therefore based on BJA results.

### Per case and clinical impact analysis

Data on antibiotic treatments for SOC- and BJA-positive cases are shown in Table 1, and for the SOC-negative, but BJA-positive cases are depicted in Table 2. Nine of 27 patients with SOC-positive cultures received empiric antibiotics prior to and 26/27 after SF collection. One patient refused antibiotic treatment (case 2). Four cases initially received inadequate therapy, of which all but two were changed to adequate therapy after BJA or SOC results became available. With regard to the SOC-negative, but BJA-positive cases, four patients were started on antibiotic treatment based on the BJA results or received optimized treatment based on these results. Strikingly, overall, in 11 of 38 (28.9%) cases, BJA results led to de-escalation of antibiotic therapy to an optimal agent, or optimized therapy was immediately initiated prior to the availability of species identification and susceptibility testing by SOC diagnostics. Six of those 11 cases occurred in SOC-positive specimens. In case 33, the BJA result was available 22:11 h earlier than the SOC and optimization occurred around 2 h after BJA availability. In case 44, BJA results were 50:21 h more quickly available than SOC, with a time to optimization of 4:41 h after BJA availability. In cases 78, 125, 163, and 165 the optimization was performed based on BJA results after 4 h, 20 h, 1.5 h, and 7.5 h, after availability, respectively.

## DISCUSSION

In JI, optimal patient management, including surgical strategies and antibiotic treatment, depends critically on the identification of the causative pathogen. Numerous studies have shown that current microbiological approaches based on culture techniques have inherent limitations in terms of speed and diagnostic accuracy (8, 10–14). Therefore, sensitive, culture-independent pathogen identification using molecular techniques has the potential to improve the quality of care for JI. Here, we prospectively evaluated the accuracy of the BJA at a university hospital center in Germany and collected real-world data on total turnaround time and impact on clinical decision-making. We observed high sensitivity and specificity for on panel pathogens (96.3% and 97.8%, respectively) and a coverage rate of 92.6%, which is in line with other previously published studies (13, 20–24). The inability of the BJA to detect *Cutibacterium spp*. and CoNS is a major drawback of the system and limits its use in PJI. Surprisingly, in contrast to a previous report from our center, none of the 15/24 culture-proven PJI were caused by *Cutibacterium spp*., CoNS, or other off-panel species, and in fact, the use of BJA in PJI in the cohort studied increased pathogen recovery by 16.7%. Although the unusual pathogen distribution in our PJI cases is most likely due to the fact that our university medical center only treats very selected PJI cases (e.g., severe underlying medical conditions, complicated courses), the finding highlights that BJA may be useful in selected PJI patient groups [e.g. late acute PJI; (20, 25, 26)]. In fact, not a single infection was identified in the study population that was only SOC-positive and BJA-negative, as would be the case with off-panel pathogens, for example.

Pathogen identification in bacterial arthritis using molecular methods hypothetically offers a faster time to result (13). The anticipated impact on time to result was confirmed here, as in culture-positive cases, the turnaround time of BJA was around 21 h shorter when compared with the SOC process. A previous study from our institute, where BJA was performed retrospectively and therefore the clinician could not act on the result, identified 42.1% of cases where BJA could have changed the antibiotic treatment (13). In 22.2% of SOC-positive cases and 45.5% of SOC-negative, but BJA-positive, cases, initial antibiotic therapy was initiated or optimized based on BJA results, and this evidence indicates that BJA has the potential to allow for faster initiation of an appropriate antibiotic therapy. Delayed introduction of active antibiotics has been shown to overall prolong antibiotic therapy, prolong the length of hospital stay, and lead to higher costs (27). Future studies will have to test the hypothesis that use of the BJA directly impacts patient outcome parameters.

It is well documented that culture-based methods tend to fail if antibiotics have been applied prior to SF aspiration (12, 13, 28). This is illustrated by the fact that in eight of 11 SOC-negative but BJA-positive cases, systemic antibiotics were administered prior to SF collection. Taken together, the performance data suggest that BJA has the ability to reduce the number of etiologically unclear cases of infectious arthritis and thus represents an important addition to the standard approach to SF diagnosis.

The use of molecular techniques for pathogen detection, however, is associated with significantly increased costs. To save resources, it is thus necessary to employ assays only if a clear clinical benefit can be expected. In this study, all SF sent for microbiological analysis were tested using BJA, including cases in which an infectious disease was rather unlikely (e.g., in degenerative joint diseases). The inclusion of 81 cases with non-inflammatory, non-infectious diseases resulted in a low pre-test probability of positivity and, therefore, a low probability that the test results would provide useful information for clinical patient management. Therefore, it is evident that the use of the BJA must be accompanied by the implementation of appropriate diagnostic stewardship measures to ensure that there is a clear indication of its effective use. This can be achieved, for example, through healthcare provider training, appropriate electronic order entry systems, and ongoing monitoring of test ordering by a laboratory utilization committee (29, 30). This could be also accompanied by a lab-based algorithm, triggering reflex testing of sterile or inconclusive samples, with high suspicion of infection (31). The use of molecular panel analysis in the diagnosis of meningitis cases has shown that in-lab reflex testing strategies are a particularly effective tool for increasing the pre-test probability of pathogen detection (32). Here, the performance of costly analyses is linked to specific, objectifiable rule-in criteria, that is, CSF pleocytosis (32, 33). In the context of the diagnosis of infectious arthritis, the determination of the total number of SF cells could offer a similar possibility (34). Above a defined cutoff (e.g., 50,000–100,00 cells/µL SF) and, if necessary, other parameters (e.g., positive leukocyte esterase, % polymorphic neutrophils, alpha-defensin), the laboratory could pro-actively and independently of the clinician decide to use the BJA (34). Rigorous selection of specimens to be tested with molecular assays is even more warranted, as false-positive results can occur and lead to inappropriate treatment decisions.

This study has several limitations, for example, the single-center study design, and, owing to a low prevalence of multidrug-resistant organisms, non-inclusion of cases related to infections with resistant isolates. Furthermore, SOC at that time included only 48 h of growth under anaerobic conditions, and it cannot be excluded that prolonged anaerobic incubation might have resulted in a higher SOC positivity rate. Another important limitation is the non-integration of BJA testing into a dedicated antimicrobial stewardship program (ASP). Previous reports showed that rapid diagnostics, for example, in blood culture processing, are most effective if results are communicated through ASP structures, ensuring immediate and appropriate translation of laboratory findings into clinical action (35). In our setting, changes in clinical management were at the discretion of the treating clinician, and it seems possible that more rigorous and structured, ASP-driven communication of test results might have further improved the impact of the BJA on the selection of antibiotics (30).

In conclusion, the BJA proved to be an accurate diagnostic tool in real-life settings, especially in acute NJIs. The assay proved to be of particularly useful in patients, who received antibiotic therapy prior to SF aspiration, and fast TTAT allowed for the timely start of appropriate antibiotic therapies or timely de-escalation. Future studies will need to establish decision rules for optimal usage of the assay and analyze patient outcomes if the BJA is integrated into ASPs.

## SUPPLEMENTAL MATERIAL

Table S1 spectrum.02239-24-s0001.xlsx Spectrum of species and resistance determinants detected by the Biofire JI panel.

Table S2 spectrum.02239-24-s0002.xlsx Summary of patient characteristics.

Table S3 spectrum.02239-24-s0003.xlsx Per specimen of joint type, clinical classification and results of SOC and Biofire JI assay.

ASM does not own the copyrights to Supplemental Material that may be linked to, or accessed through, an article. The authors have granted ASM a non-exclusive, world-wide license to publish the Supplemental Material files. Please contact the corresponding author directly for reuse.
